# Magnetic Resonance vs. Intraoral Ultrasonography in the Preoperative Assessment of Oral Squamous Cell Carcinoma: A Systematic Review and Meta-Analysis

**DOI:** 10.3389/fonc.2019.01571

**Published:** 2020-02-04

**Authors:** Filippo Marchi, Marta Filauro, Andrea Iandelli, Andrea Luigi Camillo Carobbio, Francesco Mazzola, Gregorio Santori, Giampiero Parrinello, Frank Rikky Mauritz Canevari, Cesare Piazza, Giorgio Peretti

**Affiliations:** ^1^IRCCS Ospedale Policlinico San Martino, Genoa, Italy; ^2^Department of Experimental Medicine (DIMES), University of Genoa, Genoa, Italy; ^3^Department of Surgical Sciences and Integrated Diagnostics (DISC), University of Genoa, Genoa, Italy; ^4^Department of Otorhinolaryngology, Maxillofacial, and Thyroid Surgery, Fondazione IRCCS, National Cancer Institute of Milan, University of Milan, Milan, Italy

**Keywords:** oral cavity, squamous cell cancer (SCC), magnetic resonance imaging (MRI), ultrasound, depth of invasion (DOI), tumor thickness

## Abstract

**Background:** Preoperative assessment is critical to decide the most adequate surgical strategy for oral squamous cell carcinoma (SCC). Magnetic resonance (MR) and intraoral ultrasonography (US) have been reported to be of great value for preoperative estimation of depth of invasion (DOI) and/or tumor thickness (TT). This review aims to analyze the accuracy of MR and intraoral US in determining DOI/TT in oral SCC, by assuming histological evaluation as the reference method.

**Methods:** The procedure was conducted following the modified 2009 Preferred Reporting Items for Systematic Reviews and Meta-Analyses (PRISMA) statement. We performed a systematic search of papers on PubMed, Scopus, Web of Science, and Cochrane Library databases until July 31st, 2019. For quantitative synthesis, we included nine studies (487 patients) focused on MR, and 12 (520 patients) focused on intraoral US. The Pearson correlation coefficient (*r*) between DOI/TT evaluated with MR or intraoral US was assumed as effect size. A meta-analysis (MA) for each study group (MR and US) was performed by using the random-effects models with the DerSimonian–Laird estimator and *r*-to-*z* transformation.

**Results:** In the MA for MR studies, a high heterogeneity was found (*I*^2^ = 94.84%; *Q* = 154.915, *P* < 0.001). No significant risk of bias occurred by evaluating funnel plot asymmetry (*P* = 0.563). The pooled (overall) *r* of the MR studies was 0.87 (95% CI from 0.82 to 0.92), whereas the pooled *r*-to-*z* transformed was 1.44 (95% CI from 1.02 to 1.85). In the MA for US studies a high heterogeneity was found (*I*^2^ = 93.56%; *Q* = 170.884, *P* < 0.001). However, no significant risk of bias occurred (*P* = 0.779). The pooled *r* of the US studies was 0.96 (95% CI from 0.94 to 0.97), whereas the pooled *r*-to-*z* transformed was 1.76 (95% CI from 1.39 to 2.13). These outputs were confirmed in additional MA performed by enrolling only MR (*n* = 8) and US (*n* = 11) studies that evaluated TT.

**Conclusions:** MR and intraoral US seem to be promising approaches for preoperative assessment of DOI/TT in oral SCC. Remarkably, a higher pooled *r* and *r*-to-*z* transformed were observed in the intraoral US studies, suggesting that this approach could be more closely related to histopathological findings.

## Introduction

Head and neck tumors are the sixth most common malignancies worldwide, of which oral cavity accounts for one-third (1). Most of these lesions are represented by squamous cell carcinoma (SCC), and their commonest site of presentation is the mobile tongue, followed by lips vermilion, floor of the mouth, and buccal mucosa. The vast majority of patients are male, heavy smokers, and with a history of alcohol abuse.

The recently released 8th edition of the AJCC-UICC TNM staging system (2, 3) brought relevant changes in the T classification of oral SCC. Notably, it introduced the concept of neoplastic depth of invasion (DOI) as one of the main features to be considered in the process of tumor staging. DOI is defined as “the deepest invasion of tumor in the tissue from the mucosal surface or from a theoretical reconstructed normal mucosal line” (3). It therefore differs in a fundamental way from tumor thickness (TT), since the latter is defined as the distance of the tumor surface from the deepest level of invasion (4). As a consequence of this, DOI can be significantly lower than TT in exophytic lesions, while it tends to be higher in ulcerated ones. On the other hand, the two measures may overlap each other in case of substantially flat tumors.

The intrinsic value of DOI for understanding the biologic behavior of a given oral SCC is of paramount importance in predicting regional lymph node metastasis. That being the case, identifying which radiological examination performs best in giving a precise preoperative assessment of DOI is of the greatest value. In fact, even though definitive DOI estimation will derive only from measures done on the formalin-fixed specimen (*per se* also subjected to unpredictable variations in terms of shrinkage due to the elastic properties of soft tissues and the process of chemical fixation itself), having a precise preoperative evaluation of this parameter allows the surgeon to accurately plan the resection, as well as simultaneous prophylactic neck dissection (for oral SCC with DOI > 4 mm). Moreover, the concept of adequate surgery within three-dimensional free resection margins cannot be overemphasized: in fact, it also represents an essential treatment-related prognosticator in terms of local, loco-regional control, and disease-free survival (5).

The standard diagnostic workup for oral SCC includes head and neck magnetic resonance (MR) and chest computed tomography (CT) scan in order to acquire a comprehensive TNM staging of the lesion. In the last decades, data from the literature showed a promising role of intraoral ultrasonography (US) in the preoperative evaluation of TT and/or DOI of a given lesion (6). The aim of the present review and meta-analysis was therefore to identify the best radiological examination (MR vs. intraoral US) to be offered to patients affected by oral SCC, to assess tumor clinical staging, and, consequently, to tailor the best surgical treatment in terms of oncological outcomes and minor ensuing comorbidities.

## Materials and Methods

We adhered to the *Preferred Reporting Items for Systematic Reviews and Meta-Analyses (PRISMA) guidelines* (7). The PRISMA checklist for this study is reported in Table S1. We registered our protocol with the *International Prospective Register of Systematic Reviews* (PROSPERO) (PROSPERO registration number: 102553).

### Search Strategy

We performed a systematic search on PubMed, Scopus, Web of Science, and Cochrane Library databases of papers published from January 1st, 1978, until July 31st, 2019, with the combined query “(MR OR magnetic resonance) and (oral cavity OR head and neck) and (cancer OR tumor OR carcinoma) and (thickness OR depth of invasion OR depth of infiltration)” and “(US OR ultrasonography) and (oral cavity OR head and neck) and (cancer OR tumor OR carcinoma) and (thickness OR depth of invasion OR depth of infiltration),” and their synonyms in the title and abstract fields (more details about searching and queries are reported in Table S2). Subsequently, the full text of relevant studies was screened for final selection. The references in all studies included were also searched to identify further potentially eligible studies. When multiple publications of the same research group/center described case series potentially overlapping, we used the more recent publication, if eligible.

### Eligibility Assessment

All studies identified by the initial literature search were reviewed independently by three authors (MF, FM, and ALCC). All titles and abstracts were assessed and, when in doubt, the full text scrutinized. If a dispute remained, this was resolved by one of the senior authors (GP). Inclusion criteria for evaluation of tumor DOI or TT with MR were: patients affected by oral SCC confirmed at histopathology, preoperative measurement of DOI and/or TT by MR, and comparison with histopathological DOI and/or TT. Inclusion criteria for evaluation of tumor DOI with US were: patients affected by histopathologically confirmed oral SCC, preoperative or intraoperative measurement of DOI and/or TT performed by intraoral US, and comparison with histopathological DOI and/or TT. Exclusion criteria were: duplicated articles, book chapters, case reports, poster presentations, articles analyzing different head and neck malignancies or other subsites rather than oral cavity, and articles in a language other than English (7).

### Appraisal of Study Quality

Risk of bias of each included study was assessed by using the *Quality Assessment of Diagnostic Accuracy Studies* (QUADAS)-2 tool (8). The overall quality of evidence at the outcome level was assessed according to the *Grading of Recommendations, Assessment, Development and Evaluations* (GRADE) system (9). Four reviewers were contents experts (FM, MF, AC, GP), and one reviewer (GS) was an expert statistician. The contents experts only assessed potential publications with respect to the appropriateness of the research questions. The statistician only evaluated the appropriateness of methods employed. Disagreement was resolved by consensus.

### Data Extraction

Data extraction included the following fields: study (conventionally reported with the first author), year of publication, mode of patient recruitment, country, number of patients, gender, age, tumor site, TNM staging according to the 7th edition of the AJCC-UICC staging system for oral SCC (10, 11), intraoral US device and type of probe, MR device, linear correlation between intraoral US and histopathology, and linear correlation between MR and histopathology (Table S3).

### Statistical Analysis

The statistical heterogeneity among studies was expressed as τ^2^ and estimated by Cochrane's *Q* test (11). The *I*^2^ was calculated to assess variability due to heterogeneity rather than chance (*I*^2^ ≤ 25%: low; *I*^2^ > 25% and ≤50%: moderate; *I*^2^ > 50% and ≤75%: considerable; *I*^2^ > 75%: high heterogeneity). *H*^2^ was the ratio between total and sampling variability. For both *I*^2^ and *H*^2^, the 95% confidence interval (CI) was calculated.

The meta-analysis was carried out by assuming the Pearson correlation coefficient (*r*) between MR or US and TT/DOI measurements on incisional biopsies (“gold standard”) as the effect size. Considering that in the majority of enrolled studies the TT was assessed with MR or US, we performed a further meta-analysis to evaluate only the outcomes of the TT-related studies. The DerSimonian–Laird estimator was used in the random-effects models (12) with Fisher's *r*-to-*z* transformation. Forest plots were created for each measured outcome to illustrate the effects of the different studies and the global estimation. In the random-effects models, the selected studies and their outcomes are assumed to be a random selection from a larger population of studies. The random-effects models were evaluated for each effect size without and with moderator variables; in the latter case, we obtained the corresponding mixed-effects models (one model for each moderator), where the coefficients from the fitted models estimate the relationship between the average true effect/outcome in the population of studies and the moderator variables included in the same models. The Knapp and Hartung method was used to adjust the standard errors of the estimated coefficients, which helps to account for the uncertainty in the estimate of residual heterogeneity. When moderators were included in the models, the *Q*_*E*_-test was used to evaluate residual heterogeneity.

The publication bias related to data asymmetry was estimated by funnel plots and Egger's test (13). For further evaluation of heterogeneity in each model, we evaluated also radial plots (14), normal quantile–quantile (Q-Q) plots (15), and Baujat plots (16). Statistical significance was assumed in each test with *P* < 0.05. Statistical analysis was carried out by using the R software/environment (version 3.6.1; R Foundation for Statistical Computing, Vienna, Austria), with the metafor (version 2.1-0) (17) and metacor (version 1.0-2) (18) R packages.

## Results

### Literature Search and Study Identification for MR

**Figure 1:**
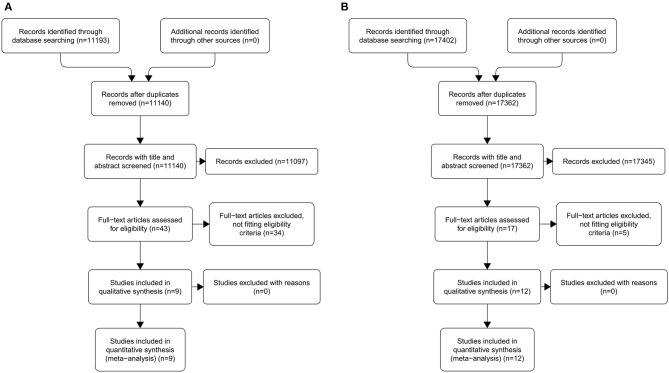
Preferred Reporting Items for Systematic Reviews and Meta-Analyses (PRISMA) flow diagram of the search results for original studies related to magnetic resonance **(A)** and ultrasonography **(B)**.

An overview of our selection process for MR-related studies is presented in Figure 1A. An initial keyword search of the listed databases identified 11,193 records. After duplicate removing and after excluded records in titles and abstracts, 43 full-text articles were assessed for eligibility (Table S4A). Of these, nine were deemed eligible for inclusion in the meta-analysis.

### Literature Search and Study Identification for Intraoral US

An overview of our selection process for US-related studies is presented in Figure 1B. An initial keyword search of the listed databases identified 17,402 records. After duplicate removing and after excluded records in titles and abstracts, 17 full-text articles were assessed for eligibility (Table S4B). Of these, 12 were deemed eligible for inclusion in the meta-analysis. The study by Yesuratnam et al. (19) was conducted performing MR and intraoral US simultaneously on each patient recruited and, for this reason, was included in both arms of the meta-analysis.

### Characteristics of Included MR Studies

The studies included in the meta-analysis are summarized in Table 1. All studies compared TT or DOI measured by MR with TT or DOI measured on histopathological slides. A total of 9 studies (n = 487 patients) was included (19–27). Sample size ranged from 18 to 102 patients. The articles were published over a period of 14 years (2003–2017). Patient recruitment ranged from 1997 to 2016. The studies showed a wide geographical distribution, the most substantial contributions coming from Asian countries (six studies). Among the participants included in the present meta-analysis, a significant male preponderance (72%) was observed. Average age was 55.5 years (range, 49–63.5). Six studies included patients affected by T1–T4 oral SCC (19–21, 23, 24, 26), one recruited T1–T2 lesions (25), one enrolled T1–T3 tumors (27), and one selected patients with advanced lesions (only T4a) (22). Nodal categories ranged from N0 to N3. The most frequently analyzed oral cavity subsite was the oral tongue (six studies) (19, 20, 22, 23, 25, 27), while three papers described oral SCC from different subsites (21, 24, 26). All the selected studies had good linear correlation between MR and histopathology. The Pearson *r* ranged from 0.63 to 0.99. MR was performed in six studies with a 1.5 Tesla (T) scanner, while in three with a 3.0 T scanner. Eight studies reported TT measured on MR: six of them specified also the mean value of TT (range, 8.4–25.9 mm; mean, 14.4 mm) (20–23, 26, 27), while the others only declared the Pearson *r* (19, 24). One study reported DOI measured on MR presenting only the value of Pearson *r* (25). Five studies calculated the overestimation of MR compared to the histopathology reports (mean, 2.5 mm) (19–21, 23, 27).

**Table 1:** Characteristics of included studies related to magnetic resonance.

**References**	**Nationality**	**Period**	**Study type**	**Pts (*n*)**	**Mean age**	**Gender (% of males)**	**Tumor site/subsite**	***T***	***N***	**MR machine**	**Pearson (*r*)**
Moreno et al. (20)	US	2009–2012	Prospective	25	58	68%	Tongue	T1–T4	0–2	3 T	0.84
Goel et al. (21)	India	2013–2015	Prospective	61	49	74%	Oral cavity	T1–T4	0–2	1.5 T	0.988
Yesuratnam et al. (19)	Australia	2007–2012	Prospective	81	63	58%	Tongue	T1–T4	0	3 T	0.69
Chen et al. (22)	Taiwan	2003–2006	Prospective	58	50.7	93%	Tongue	T4a	-	1.5 T	0.905
Park et al. (23)	Korea	2003–2008	Retrospective	49	54.6	78%	Tongue	T1–T4	0–2	1.5 T	0.949
Lwin et al. (24)	UK	2007–2008	Retrospective	102	59	67%	Oral cavity	T1–T4	0–2	1.5 T	0.63
Jung et al. (25)	Korea	2002–2005	Retrospective	50	52	52%	Tongue	T1–T2	0	3 T	0.851
Okura et al. (26)	Japan	1998–2007	Retrospective	43	58	67%	Oral cavity	T1–T4	0–3	1.5 T	0.86
Lam et al. (27)	Hong Kong	1997–2000	Prospective	18	63.5	88%	Tongue	T1–T3	0–1	1.5 T	0.938

*MR, magnetic resonance*.

### Characteristics of Intraoral US Included Studies

The studies included in the meta-analysis are summarized in Table 2. All studies compared TT or DOI measured preoperatively or intraoperatively by intraoral US with TT or DOI measured on histopathological slides. A total of 12 studies (*n* = 520 patients) was included (19, 28–38). The sample size ranged from 13 to 109 patients. The articles were published over a period of 17 years (2001–2018), and patient recruitment covered the period 1997–2016. The studies showed a wide geographical distribution, but also, for intraoral US, the most substantial contributions came from Asian countries, in particular, from Japan (six studies) (28, 31, 33, 35, 37, 38). There was a moderate male preponderance (59%) among the participants included in this analysis. The mean age was 60.3 years (range, 57–65). Seven studies included patients affected by T1–T4 oral SCC (19, 28, 29, 32, 36–38), and five recruited T1–T2 tumors (30, 31, 33–35). Nodal categories ranged from N0 to N3. The most frequently analyzed oral cavity subsite was the oral tongue (eight studies) (19, 28, 29, 31, 33–35, 37), while four papers described oral SCC from different subsites (30, 32, 36, 38). All the studies selected had a good linear correlation between intraoral US and histopathology. The Pearson *r* ranged from 0.83 to 0.99. The probe frequency (PF) used for intraoral US ranged from 5 to 16 MHz. Eleven studies reported TT measured by intraoral US (range, 3.8–16 mm; mean, 9.4 mm) (19, 29–38). Among these, eight also calculated the overestimation of intraoral US compared to the histopathology reports (mean, 1.7 mm) (19, 28–31, 34, 37, 38). One study reported DOI measured by intraoral US (28).

**Table 2:** Characteristic of included studies related to intraoral ultrasonography.

**References**	**Nationality**	**Period**	**Study type**	**Pts (*n*)**	**Mean age**	**Gender (% of males)**	**Tumor site/subsite**	***T***	***N***	**US probe**	**Setting**	**Pearson (*r*)**
Iida et al. (28)	Japan	2008–2015	Retrospective	56	59	61%	Tongue	1–4	0	16 MHz	Preoperative	0.86
Yesuratnam et al. (19)	Australia	2007–2012	Prospective	88	63	58%	Tongue	1–4	0	15–5 MHz	Preoperative	0.8
Chammas et al. (29)	Brazil	2006–2009	Prospective	19	60	58%	Tongue	1–4	1–3	5–10 MHz	Preoperative	0.83
Lodder et al. (30)	Netherlands	2004–2010	Retrospective	65	65	52%	Tongue/Fom	1–2	0–2	7–15 MHz	Intraoperative	0.93
Kodama et al. (31)	Japan	2005–2007	Prospective	13	61,6	62%	Tongue	1–2	0	7.5 MHz	Intraoperative	0.981
Mark Taylor et al. (32)	Canada	-	Prospective	21	65	57%	Tongue/Fom	1–4	0–2	10–12 MHz	Preoperative	0.981
Kaneoya et al. (33)	Japan	-	Prospective	48	57	56%	Tongue	1–2	0	12 MHz	Intraoperative	0.824
Baek et al. (34)	South Korea	2006–2007	Prospective	20	57	50%	Tongue	1–2	0	8–10 MHz	Intraoperative	0.744
Yamane et al. (35)	Japan	1998–2002	Prospective	109	57	70%	Tongue	1–2	0	10 MHz	Preoperative	0.985
Songra et al. (36)	United Kingdom	1997–2002	Prospective	14	-	-	Oral cavity	1–4	0–3	5–10 MHz	Preoperative	0.948
Kurokawa et al. (37)	Japan	2000–2003	Prospective	28	59,4	64%	Tongue	1–4	1–2	7.5 MHz	Preoperative	0.976
Shintani et al. (38)	Japan	-	Prospective	39	58	64%	Oral cavity	1–4	0–2	7.5 MHz	Preoperative	0.99

*US, ultrasonography. Fom, floor of mouth*.

### Quality Assessment and Risk of Bias

The risk of bias evaluated with the QUADAS-2 tool for the included MR-related studies was low in three studies (22, 23, 25), unclear in three (20, 21, 27), and high in three (19, 24, 26) (Table 3). The risk of bias evaluated with the QUADAS-2 tool for the included US-related studies was low in one study (36), unclear in seven (28, 29, 31–35), and high in four (19, 30, 37, 38) (Table 3). Following the GRADE system, the overall quality of evidence for the included studies related to MR and intraoral US was assessed as very low (Tables S5A,B).

**Table 3:** Application of the quality assessment of diagnostic accuracy studies (QUADAS)-2 for each included study.

**Study**	**Risk of bias**				**Applicability concerns**		
	**Patient selection**	**Index test**	**Reference standard**	**Flow and timing**	**Patient selection**	**Index test**	**Reference standard**
**MR**							
Moreno et al. (20)	?	L	L	L	?	L	L
Goel et al. (21)	?	L	L	L	?	L	L
Yesuratnam et al. (19)	H	L	L	L	L	L	L
Chen et al. (22)	L	L	L	L	L	L	L
Park et al. (23)	L	L	L	L	L	L	L
Lwin et al. (24)	H	L	L	L	H	L	L
Jung et al. (25)	L	L	L	L	L	L	L
Okura et al. (26)	?	?	L	H	?	?	L
Lam et al. (27)	L	?	L	L	L	?	L
**US**							
Iida et al. (28)	L	?	L	?	L	?	L
Yesuratnam et al. (19)	H	L	L	L	L	L	L
Chammas et al. (29)	?	L	L	L	?	L	L
Lodder et al. (30)	L	H	L	H	L	H	L
Kodama et al. (31)	?	L	L	L	?	L	L
Mark Taylor et al. (32)	?	L	L	?	?	L	L
Kaneoya et al. (33)	?	L	L	?	?	L	L
Baek et al. (34)	L	?	L	L	L	?	L
Yamane et al. (35)	L	?	L	L	L	?	L
Songra et al. (36)	L	L	L	L	L	L	L
Kurokawa et al. (37)	H	?	L	L	H	?	L
Shintani et al. (38)	H	?	L	?	H	?	L

*MR, magnetic resonance; US, ultrasonography H, high risk; L, low risk; ?, uncertain*.

### Meta-Analysis for Studies Related to MR

**Figure 2:**
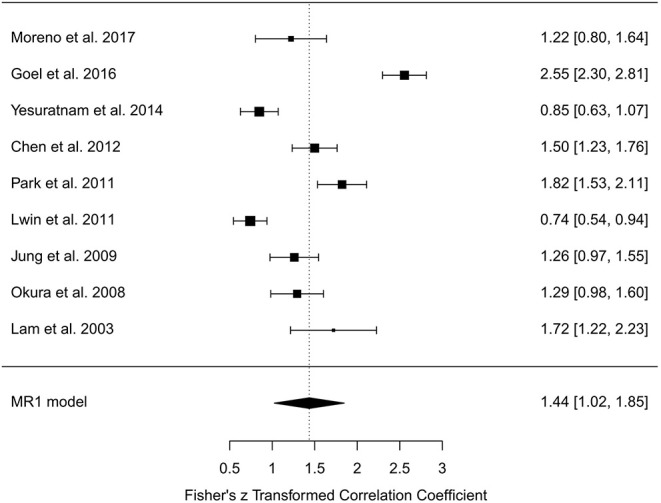
Forest plot for *r*-to-*z* transformation in the random-effects model with included studies related to magnetic resonance (MR1 model).

**Figure 3:**
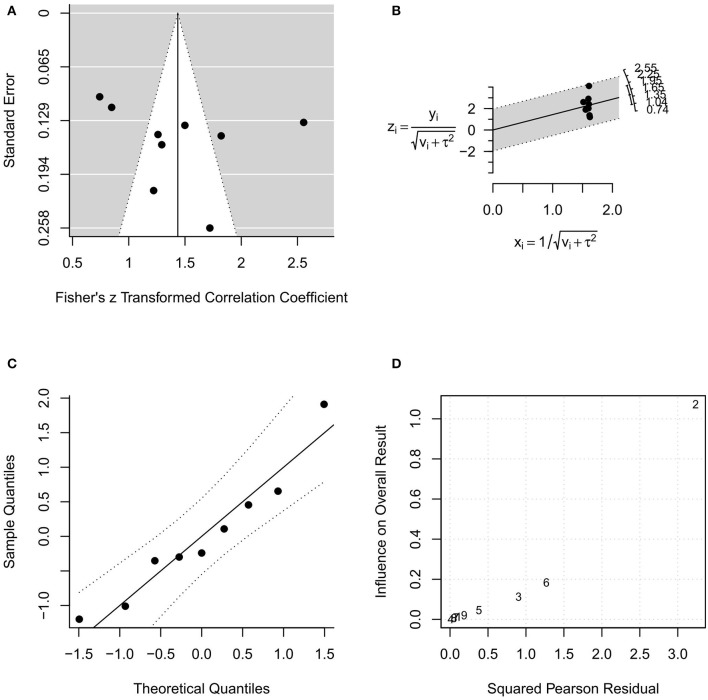
Plots for evaluating publication bias and heterogeneity in the random-effects model with included studies related to magnetic resonance (MR1 model). **(A)** funnel plot; **(B)** radial plot; **(C)** normal quantile–quantile plot; **(D)** Baujat plot.

In the random-effects model with all MR studies (*n* = 9) included (MR1 model), the *I*^2^ was 94.84% (95% CI, 85.67–98.18), and the *H*^2^ was 19.36 (95% CI, 6.98–55.06), with *Q* = 154.915 (*P* < 0.001). The pooled *r* was 0.87 (95% CI, 0.82–0.92), whereas the pooled *r*-to-*z* transformed was 1.44 (95% CI, 1.02–1.85). The MR1 model resulted as statistically significant (*P* < 0.001). The forest plot of the MR1 model is reported in Figure 2, while the plots for evaluating publication bias and heterogeneity are shown in Figure 3. Egger's test for funnel plot asymmetry (Figure 3A) did not reach statistical significance (*P* = 0.563), while all studies remained within the confidence region of the Q-Q plot (Figure 3B). The Baujat plot showed that the study of Goel et al. (21) provided the greatest contribution to heterogeneity (Figure 3D). By entering the main variables (patient mean age and sex, tumor site, T, N, M, TT/DOI) as moderators in the MR1 model, only patient mean age reached statistical significance (*P* = 0.04).

In the random-effects model with only MR studies (*n* = 8) that evaluated TT (MR2 model), the *I*^2^ was 95.47% (95% CI, 86.74–98.57), and the *H*^2^ was 22.08 (95% CI, 7.54–70.09), with *Q* = 154.556 (*P* < 0.001). The pooled *r* was 0.87 (95% CI, 0.82–0.93), whereas the pooled *r*-to-*z* transformed was 1.46 (95% CI, 0.99–1.92). The MR2 model resulted as statistically significant (*P* < 0.001). The forest plot of the MR2 model is reported in Figure S1, while the plots for evaluating publication bias and heterogeneity are shown in Figure S2. Also in the RM2 model, Egger's test for funnel plot asymmetry (Figure S2A) did not reach statistical significance (*P* = 0.601), while the study of Goel et al. (21) provided the greatest contribution to heterogeneity (Figure S2D). By entering moderators in the MR2 model as described above, only patient mean age reached statistical significance (*P* = 0.019).

### Meta-Analysis for Studies Related to Intraoral US

**Figure 4:**
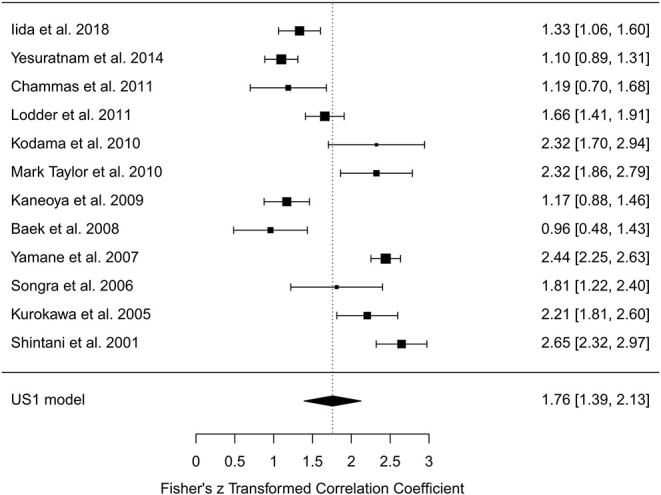
Forest plot for *r*-to-*z* transformation in the random-effects model with included studies related to intraoral ultrasonography (US1 model).

**Figure 5:**
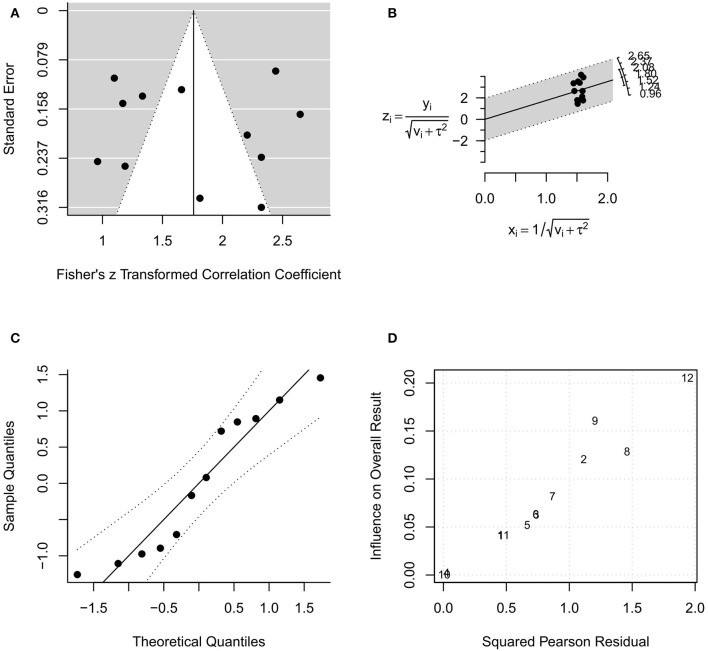
Plots for evaluating publication bias and heterogeneity in the random-effects model with included studies related to intraoral ultrasonography (US1 model). **(A)** funnel plot; **(B)** radial plot; **(C)** normal quantile–quantile plot; **(D)** Baujat plot.

In the random-effects model with all intraoral US studies (*n* = 12) included (US1 model), the *I*^2^ was 93.56% (95% CI, 85.03–97.50), and the *H*^2^ was 15.53 (95% CI, 6.68–40.06), with *Q* = 170.884 (*P* < 0.001). The pooled *r* was 0.96 (95% CI, 0.94–0.97), whereas the pooled *r*-to-*z* transformed was 1.76 (95% CI, 1.39–2.13). The US1 model resulted as statistically significant (*P* < 0.001). The forest plot of the US1 model is reported in Figure 4, while the plots for evaluating publication bias and heterogeneity are shown in Figure 5. Egger's test for funnel plot asymmetry (Figure 5A) did not reach statistical significance (*P* = 0.779), while all studies remained within the confidence region of the Q-Q plot (Figure 5B). The Baujat plot showed that the studies of Baek et al. (34), Yamane et al. (35), and Shintani et al. (38) provided the greatest contribution to heterogeneity (Figure 5D). By entering the main variables (patient mean age and sex, tumor site, T, N, M, TT/DOI, probe) as moderators in the US1 model, patient sex (*P* = 0.006) and probes at 12 MHz (*P* = 0.005), 5–15 MHz (*P* = 0.002), 8–10 MHz (*P* = 0.003), and 5–10 MHz (*P* = 0.033) reached statistical significance.

In the random-effects model with only intraoral US studies (*n* = 11) that evaluated TT (US2 model), the *I*^2^ was 93.76% (95% CI, 84.69–97.67), and the *H*^2^ was 16.03 (95% CI, 6.53–42.95), with *Q* = 160.321 (*P* < 0.001). The pooled *r* was 0.96 (95% CI, 0.95–0.98), whereas the pooled *r*-to-*z* transformed was 1.80 (95% CI, 1.40–2.20). The US2 model resulted as statistically significant (*P* < 0.001). The forest plot of the US1 model is reported in Figure S3, while the plots for evaluating publication bias and heterogeneity are shown in Figure S4. Also in the US2 model, Egger's test for funnel plot asymmetry (Figure S4A) did not reach statistical significance (*P* = 0.892), while the studies of Yesuratnam et al. (19), Baek et al. (34), and Shintani et al. (38) provided the greatest contribution to heterogeneity (Figure S4D). By entering moderators in the US2 model as described above, patient sex (*P* = 0.005) and probes at 12 MHz (*P* = 0.005), 5–15 MHz (*P* = 0.002), 8–10 MHz (*P* = 0.003), and 5–10 MHz (*P* = 0.033) reached statistical significance.

## Discussion

To the best of our knowledge, this is the first systematic review with meta-analysis of the literature that compares the accuracy of MR and intraoral US in the measurement of TT and/or DOI in oral SCC. Nevertheless, this topic merits considerable attention due to the well-recognized role of DOI as a prognosticator in terms of oncological outcomes during and after management of oral cancer. To choose the most appropriate therapy for such tumors, in fact, two fundamental issues must be carefully taken into account: the longitudinal extension in depth and involvement of the extrinsic tongue musculature. Before the introduction in clinical practice of the 8th edition of the TNM staging system, TT was the most common measure used to assess the longitudinal extension in depth of oral cavity tumors. Different authors recently underlined the correlation between DOI and TT, showing that, even though the two concepts are quite different, the T category and TNM stage prognostic performances within the 8th edition of the TNM are similar regardless of whether DOI or TT is used as a T-category modifier (39). Furthermore, it has been demonstrated that both TT and DOI are highly correlated with nodal risk but with different cutoff points for prediction (40). However, DOI is nowadays considered as the most reliable parameter, better correlating with the risk of nodal metastasis and patient prognosis. In spite of this, authors enrolled in the present meta-analysis articles referring to both DOI and TT because the aim of the study was to investigate the tools' accuracy in measuring linear spatial dimensions in relation to the histopathological standards, rather than attributing any oncological value to any of these two variables.

The second important detail to be evaluated is the possible involvement of the extrinsic tongue muscles that are in close relationship (in the range of a few millimeters) with the mucosa overlying the floor of the mouth and the posterior-lateral portion of the lingual body. In such a case, the risk of in-transit metastases along the T-N tract is consistently high (41). The T-N tract is represented by the sublingual and submandibular glands, mylohyoid muscle, lingual nerve, artery and vein, and all the stromal tissue, and lingual and sublingual lymph nodes of the compartment: all these structures are anatomically present between the tongue (T) and the first lymph node level (N) in the neck. The T-N tract plays an important role in prognosis and survival in patients with tongue and floor-of-the mouth cancer (42). Hence we can assume that when extrinsic muscles are not involved by the tumor, usually for T1–T2 with DOI < 10 mm (3), the lesion can be safely removed transorally. On the other hand, in more advanced stages, the paradigm shift from circumferential and/or cuneiform to longitudinal compartmental resection allows obtaining the best loco-regional control (41, 43). DOI, particularly for early-intermediate lesions (cT1–T2N0), is crucial in deciding whether to perform a simultaneous neck dissection or defer it after formal histopathological evaluation raising the doubt of a higher risk for occult nodal metastasis (18, 19). It is well-established, in fact, that DOI is strictly related to the probability of having occult nodal metastases in regional lymph nodes. Mohit-Tabatabai et al. (44) and Spiro et al. (45) first applied Breslow's hypothesis (46) regarding an existent link between lymph node involvement and DOI in oral SCC. However, till now, controversy still persists about the optimal DOI cutoff for a clinically relevant risk of occult nodal disease. Several studies in the literature concluded that such an optimal cutoff point could be set at 4 mm: therefore, in cN0 patients with DOI < 4 mm, an elective neck dissection could in theory be safely spared (20, 24, 26, 27). Even though histological determination of DOI is so far the gold standard in the decision-making process of performing or not performing prophylactic neck dissection for early oral tongue SCC, its accurate preoperative measurement allows performing such an elective surgical procedure simultaneously with tumor excision only in selected cases, thus reducing the number of undue overtreatments.

Nowadays, MR remains the most precise tool for oral SCC loco-regional staging, with a reported sensitivity of 94% (47). The best MR sequence to define DOI is considered to be T1-weighted contrast-enhanced. On the other hand, a T2-weighted sequence overestimates tumor volume, since peritumoral inflammation and edema cannot be distinguished from the actual tumor. In fact, tumor, peritumoral edema, and inflamed tissues show the same T2 repetition times, while T1-weighted sequence allows a more affordable distinction between them.

Singh et al. (48) reported good agreement (k value of 0.790) for T staging between MR and definitive histopathological evaluation, with the final pT category changed in only 14% of their patients. Furthermore, Moreno et al. (20) recently reported their experience in using 3.0 T MR for oral tongue carcinoma staging: the increased signal-to-noise ratio and larger susceptibility resulted in higher spatial resolution, leading to improved imaging, and diagnostic strength. The primary objective of their study was to evaluate the efficacy of the 3.0 T MR in predicting TT of oral SCC compared with histological measures. The secondary end point was to compare radiographic and pathological nodal staging, evaluating the relationship between TT of oral SCC and the presence of cervical lymph node metastasis, and to assess the capacity of the 3.0 T machine to predict extracapsular extension. In their series, TT at MR was always significantly higher than the histological value, with a Pearson correlation coefficient of 0.81. By contrast, 3.0 T MR seems to have a higher sensitivity (83.3%), specificity (81.8%), and accuracy (82.3%) than the 1.5 T MR in predicting nodal metastasis.

Baek et al. (34) recently confirmed the usefulness of intraoral US in predicting pathologic TT of oral tongue SCC. Moreover, they underlined how both CT and MR have some limitations in evaluation of tongue cancers, with MR providing superior information over CT in what concerns soft tissues. Yesuratman et al. (19) found that preoperative intraoral US demonstrated a high correlation with histopathological TT (*r* = 0.80), while MR only a moderate one (*r* = 0.69). Furthermore, they identified biopsy performed before imaging as a significant confounding factor in reducing the accuracy of preoperative imaging. Intraoral US and MR were, in fact, unable to differentiate post-biopsy hematomas from squamous dysplasia and/or invasive SCC. In this scenario, intraoral US seems especially useful in discerning structures of crucial soft tissues. The possibility to move the probe along different planes, even through the skin of the face if needed, and to ask the patient to protrude and move the tongue or swollen cheek, are simple but effective tools used to evaluate thin muscles and buccal fat layers. However, due to the heterogeneity of the instrumentation used in the selected studies, the best probe, as well as the ideal PF and modality to be used for the examination, is still to be defined. In this respect, Iida and coworkers (28) reported a T-shape linear probe fitted with a rubber sheath and filled with water as an ideal tool to preoperatively measure DOI. The aforementioned features, however, allow the surgeon to better delineate the actual three-dimensional extension of the disease, especially considering that intraoral US may be repeated in the operatory room, just before surgery; it also may be used intraoperatively to check the resection margins and even *ex vivo* on the resected specimen (34, 36, 49).

The main limitation of intraoral US is the impossibility of reaching some oral subsites and the impediment given by bony structures: in fact, lesions located in the posterior third of the mobile tongue are not easily accessible to a perpendicular evaluation. Furthermore, when performing intraoral US, the probe should be kept in tight contact with the organ to be evaluated to improve the interface between them, but without exerting too much pressure that could deform or modify the shape of the neoplasm itself. As an intrinsic limit of this technique, intraoral US is a strongly operator-dependent live examination: as a consequence, different radiologists with variable experiences may report different information. Moreover, the same exam cannot be reassessed by a second operator at a later time.

Both MR and intraoral US were shown herein to have good correlation with histopathological findings. Nonetheless, the higher pooled *r* was seen in the intraoral US studies (0.96 vs. 0.87), even though more research is needed to standardize the optimal PF. On the top of this, however, intraoral US was a better preoperative and intraoperative predictor of TT and DOI, particularly in early SCC located in the anterior part of the oral cavity.

### Limitations of the Meta-Analysis

Although we performed extensive analyses and diagnostic plots by using an open source environment, making our study potentially reproducible (50), this review is affected by several limitations. Potential weakness may have affected the review process (unintentional omission of papers and/or impossibility to retrieve some). Although we did not find significant funnel plot asymmetry for potential publication bias, by applying the QUADAS-2, a high risk of bias occurred in three MR-related studies (19, 24, 26) and in four US-related studies (19, 30, 37, 38), with an overall low quality assessment for both MR- and US-related studies following the GRADE system. Moreover, Cochrane's *Q* was significant in all models evaluated in the meta-analysis, suggesting a high heterogeneity of the pooled data. These findings are likely to reflect several methodological “gray zones” of the selected studies. Not all studies clearly specified if a double-blinded exam evaluation process had been performed. The period of enrollment ranged from 3 to 10 years among MR studies, and from 2 to 7 years for those concerning intraoral US. The probability that radiological techniques and operators may have changed during this time frame is proportional to the duration of the enrollment period. The number of cases analyzed by each paper significantly diverged from 18 to 102 for MR and from 13 to 109 for intraoral US. The process of patient selection significantly differed among the studies: six of nine MR publications took into account T1–T4 patients (19–21, 23, 24, 26), one study T1–T3 (27), one only T1–T2 (25), and the last only T4a patients (22). Among the 12 intraoral US papers, seven studies considered T1–T4 patients (19, 28, 29, 32, 36–38), while five early-stage tumors (T1–T2) only (30, 31, 33–35). In this regard, a larger discrepancy in measurements is more probable to happen in T4 patients compared to flatter and smaller lesions. Execution modality and evaluation of the diagnostic exams were not always uniform among the considered papers: different equipment was used; the timing between imaging and histopathological examination, as well as between preoperative biopsy and imaging, was inconstant or unexplained. Furthermore, only one MR and one intraoral US study considered DOI as a benchmark with histopathology. Finally, although we conducted a meta-regression to detect whether clinical variables influenced the results, some variables could not be quantitatively detected. These included the diagnostic ability of radiologists and individual, unreported clinical variables. Inherent diversity among studies, small sample sizes, and other unpredictable biases are all possible limitations.

## Conclusions

Guidelines for diagnosis, treatment, and follow-up of head and neck SCC consider MR as the preferred staging method for each site and subsite except the larynx and hypopharynx (51). In fact, it offers the best discrimination of soft tissues, contouring of tumor borders, neoplastic extension, and identification of intracranial and/or perineural spreads, and also enables the radiologist to analyze intratumoral vascularization. On the other hand, the limits of MR are mainly represented by the impossibility to perform the examination in patients who are claustrophobic, non-compliant, or with metallic prostheses and pacemakers. Also, significant artifacts due to metallic implants or other fixed prostheses may significantly hamper the intraoral imaging quality. By contrast, intraoral US is definitely faster, less invasive, and cost-effective, and requires less patient compliance, and its high-resolution allows us to better define TT and DOI in early-stage tumors of the mobile tongue and buccal mucosa. However, it remains highly operator-dependent and difficult to use for lesions in close continuity with bony structures or located in the posterior part of the oral cavity.

Considering the limitations of the studies included and the retrospective nature intrinsic to any meta-analysis, further prospective comparisons between intraoral US and MR in the same cohort of patients are strongly recommended. This will allow the identification of the best imaging technique to be specifically used in early and advanced oral SCC, possibly with a parallel reduction in terms of patient selection biases. Nonetheless, from our data, it is possible to determine the non-inferiority of intraoral US in comparison with MR. This will allow clinicians to use a less expensive and faster tool in the preoperative diagnostic workup of selected oral SCC, as well as giving adjunctive information in case of doubtful results after MR.

## Data Availability Statement

All datasets generated for this study are included in the article/Supplementary Material.

## Author Contributions

FMar and MF: study concepts and study design. FMar, MF, AC, AI, and FMaz: data acquisition. FMaz, GPa, and FC: quality control of data and algorithms. GS: data analysis and interpretation and statistical analysis. FMar, MF, AC, and AI: manuscript preparation and manuscript editing. CP and GPe: manuscript review. All the authors have made a significant contribution to this manuscript, have seen and approved the final manuscript, and have agreed to its submission to the Frontiers in Oncology Journal.

### Conflict of Interest

The authors declare that the research was conducted in the absence of any commercial or financial relationships that could be construed as a potential conflict of interest.
