# Supplementary material for: Magnetic Resonance vs. Intraoral Ultrasonography in the Preoperative Assessment of Oral Squamous Cell Carcinoma: A Systematic Review and Meta-Analysis
Source: Front Oncol. 2020 Feb 4;9:1571. doi: 10.3389/fonc.2019.01571 (PMC7010633; doi:10.3389/fonc.2019.01571)
Supplement: Supplementary file 1 [file Data_Sheet_1.pdf]

ELECTRONIC SUPPLEMENTARY MATERIAL

Magnetic resonance versus intraoral ultrasonography in the preoperative assessment of oral squamous cell carcinoma: A systematic review and meta-analysis

Filippo Marchi, MD;[1†] Marta Filauro, MD;[1,2†] Andrea Iandelli, MD;[1] Andrea Luigi Camillo Carobbio, MD;[1] Francesco Mazzola, MD;[1] Gregorio Santori, MD, PhD;[3] Giampiero Parrinello, MD;[1] Frank Rikky Mauritz Canevari, MD;[1,3] Cesare Piazza, MD;[4] Giorgio Peretti, MD.[1,3]

[1] IRCCS Ospedale Policlinico San Martino, Genoa, Italy

[2] Department of Experimental Medicine (DIMES), University of Genoa, Genoa, Italy

[3] Department of Surgical Sciences and Integrated Diagnostics (DISC), University of Genoa, Genoa, Italy

[4] Department of Otorhinolaryngology, Maxillofacial, and Thyroid Surgery, Fondazione IRCCS, National Cancer Institute of Milan, University of Milan, Milan, Italy

[†] These authors have equally contributed to this work.

Table of Contents

ESM Table 1. PRISMA checklist (pp. 2-6).

ESM Table 2. Full search strategy (p. 7).

ESM Table 3. Extracted data in each study assessed for eligibility (p. 8).

ESM Table 4A. Full text magnetic resonance-related articles excluded, not fitting eligibility criteria (p. 9).

ESM Table 4B. Full text intraoral ultrasonography-related articles excluded, not fitting eligibility criteria (p. 10).

ESM Table 5A. Overall quality assessment for the magnetic resonance-related studies included in the meta-analysis, following the GRADE system (p. 11).

ESM Table 5B. Overall quality assessment for the intraoral ultrasonography-related studies included in the meta-analysis, following the GRADE system (p. 12).

ESM Figure 1. Forest plot for *r*-to-*z* transformation in the random-effects model for included studies with tumor thickness evaluated by magnetic resonance (MR2 model).(p. 13).

ESM Figure 2. Plots for evaluating publication bias and heterogeneity in the random-effects model for included studies with tumor thickness evaluated by magnetic resonance (MR2 model). A: funnel plot; B: radial plot; C: normal quantile-quantile plot; D: Baujat plot (p. 14).

ESM Figure 3. Forest plot for *r*-to-*z* transformation in the random-effects model for included studies with tumor thickness evaluated by intraoral ultrasonography (US2 model).(p. 15).

ESM Figure 4. Plots for evaluating publication bias and heterogeneity in the random-effects model for included studies with tumor thickness evaluated by intraoral ultrasonography (US2 model). A: funnel plot; B: radial plot; C: normal quantile-quantile plot; D: Baujat plot (p. 16).

ESM Table 1. PRISMA Checklist.

Section/topic	#	Checklist item	Reported on page # or section
**TITLE**			
Title	1	Magnetic resonance versus intraoral ultrasonography in assessment of depth of invasion in oral squamous cell carcinoma: A systematic review and meta-analysis	1
**ABSTRACT**			
Structured summary	2	**Background**: Preoperative assessment of depth of invasion (DOI) or tumor thickness (TT) is critical to decide the most adequate surgical strategy for oral squamous cell carcinoma (SCC), and to evaluate the necessity to treat the neck. Magnetic resonance (MR) and intraoral ultrasonography (US) have been reported to be of great value for preoperative estimation of DOI and/or TT. This review aims to analyze the accuracy of MR and intraoral US in determining DOI or TT in oral SCC, by assuming histological evaluation as the reference method.<br><br>**Method**: We performed a systematic search of papers on PubMed, Scopus, Web of Science and Cochrane Library databases published until July 31st, 2019. Fifty-seven full-text articles were screened for final selection and statistical analysis. The procedure was conducted following the modified 2009 PRISMA statement. For quantitative synthesis, we included 9 studies (487 patients) focused on MR and 12 (520 patients) focused on intraoral US. The correlation coefficient (r) between TT/DOI evaluated with MR or intraoral US and histopathology was assumed as effect size. A meta-analysis (MA) for each study group (MR and US) was performed by using the random-effects models with the DerSimonian-Laird estimator and r-to-z transformation.<br><br>**Results**: In the MA for MR studies, the I2 was 94.84% and the H2 was 19.36, with Q = 154.915 (P <0.001). The pooled r was 0.87 (95% CI from 0.82 to 0.92), whereas the pooled r-to-z transformed was 1.44 (95% CI from 1.02 and 1.85). The Egger's test for funnel plot asymmetry did not reach statistical significance (P = 0.563). In the MA for US studies, the I2 was 93.56% and the H2 was 15.53, with Q = 170.884 (P <0.001). The pooled r was 0.96 (95% CI from 0.94 to 0.97) whereas the pooled r-to-z transformed was 1.76 (95% CI from 1.39 and 2.13). The Egger's test did not reach statistical significance (P = 0.779). These outputs were confirmed in additional MA performed by enrolling only MR (n = 8) and US studies (n = 11) with histological TT as gold standard.<br><br>**Conclusions**: MR and intraoral US seem to be promising approaches for preoperative assessment of DOI/TT in oral SCC, although a higher pooled *r* and *r*-to-*z* transformed were observed in the intraoral US studies (PROSPERO No. 102553).	Abstract
**INTRODUCTION**			
Rationale	3	The recently released 8th Edition of the AJCC UICC TNM staging system brought relevant changes in the T classification of oral SCC. Notably, it introduced the concept of neoplastic depth of invasion (DOI) as one of the main features to be considered in the process of tumor staging. DOI is defined as "the deepest invasion of tumor in the tissue from the mucosal surface or from a theoretical reconstructed normal mucosal line". It therefore differs in a fundamental way from tumor thickness (TT), since the latter is defined as the distance of the tumor surface from the deepest level of invasion. As a consequence of this, DOI can be significantly lower than TT in exophytic	Introduction

		lesions, while it tends to be higher in ulcerated ones. On the other hand, the two measures may overlap each other in case of substantially flat tumors.<br><br>The intrinsic value of DOI for understanding the biologic behavior of a given oral SCC is of paramount importance in predicting regional lymph nodes metastasis. That being the case, identifying which radiological examination performs best in giving a precise preoperative assessment of DOI is of greatest value. In fact, even though definitive DOI estimation will derive only from measures done on the formalin-fixed specimen (per se also subjected to unpredictable variations in terms of shrinkage due to the elastic properties of soft tissues and the process of chemical fixation itself), having a precise preoperative evaluation of this parameter allows the surgeon to accurately plan the resection (e.g. choosing between transoral resection for lesions with DOI <10 mm or major ablative surgery with flap reconstruction for tumors with DOI >10 mm), as well as simultaneous prophylactic neck dissection (for oral SCC with DOI >4 mm). Moreover, the concept of adequate surgery within three-dimensional free resection margins cannot be overemphasized: in fact, it also represents an essential treatment-related prognosticator in terms of local, loco-regional control, and disease-free survival.	
Objectives	4	The aim of the present review and meta-analysis was to identify the best radiological examination [magnetic resonance (MR) or intraoral ultrasonography (US)] to be offered to patients affected by oral SCC, to assess tumor clinical staging and, consequently, tailor the best surgical treatment in terms of oncological outcomes and minor ensuing comorbidities.	Introduction
**METHODS**			
Protocol and registration	5	PROSPERO n. 102553	Materials and Methods (MM).
Eligibility criteria	6	Inclusion criteria for evaluation of tumor DOI or TT with magnetic resonance (MR) were: patients affected by oral SCC confirmed at histopathology, preoperative measurement of DOI and/or TT by MR and comparison ~~of~~ with histopathological DOI and/or TT.<br><br>Inclusion criteria for evaluation of tumor DOI with US were: patients affected by histopathologically confirmed oral SCC, preoperative or intraoperative measurement of DOI and/or TT performed by intraoral US and comparison ~~of~~ with histopathological DOI and/or TT.	MM ("*Eligibility assessment*")
Information sources	7	We performed a systematic search on PubMed, Scopus, Web of Science and the Cochrane Library databases of papers published from January 1st, 1978 until the July 31st, 2019.	MM ("*Search strategy*"), ESM Table 2.
Search	8	"(MR OR magnetic resonance) and (oral cavity OR head and neck) and (cancer OR tumor OR carcinoma) and (thickness OR depth of invasion OR depth of infiltration)" and "(US OR ultrasonography) and (oral cavity OR head and neck) and (cancer OR tumor OR carcinoma) and (thickness OR depth of invasion OR depth of infiltration)"	MM ("*Search strategy*"), ESM Table 2.
Study selection	9	Exclusion criteria were: duplicated articles, book chapters, cases reports, poster presentations, articles analyzing different head and neck malignancies or other subsites rather than oral cavity, and articles in a language other than English	MM ("*Eligibility assessment*"), ESM Table 3.
Data collection process	10	All studies identified by the initial literature search were reviewed independently by three authors (MF, FM, and ALCC). All titles and abstracts were assessed and, when in doubt, the full text scrutinized. If a dispute remained, this was resolved by one of the senior authors (GP).	MM ("*Eligibility assessment*").
Data items	11	Study (conventionally reported with the first author surname), year of publication; Country/countries in which the study was carried out; Typer of recruitment; Percentage of male patients; Patient age reported in the study (as mean or median); Number of eligible patients; tumor site and subsites; T stage; N stage; type of MR machine utilized (3T/1.5T); type of US probe utilized (frequencies in Hz); settings of the US examination (preoperative/intraoperative); Pearson's *r*	MM ("*Data extraction*").

Risk of bias in individual studies	12	Risk of bias of each included study was assessed by using the *Quality Assessment of Diagnostic Accuracy Studies* (QUADAS)-2 tool The overall quality of evidence at the outcome level was assessed according to the *Grading of Recommendations, Assessment, Development and Evaluations* (GRADE) system.	MM ("*Appraisal of study quality*").
Summary measures	13	The meta-analysis was carried out by assuming Pearson correlation coefficient (r) between MRI or US vs. TT/DOI measurements on incisional biopsies ("gold standard") as the effect size. Considering as in the majority of enrolled studies the gold standard was TT, we performed a further meta-analysis for evaluating only the TT-studies. The DerSimonian-Laird estimator was used in the random-effects models with the Fisher's r-to-z transformation	MM ("*Statistical Analysis*").
Synthesis of results	14	No results is reported in the "Methods" section. See the "Results" section and the related checklist items 17-23.	-
Risk of bias across studies	15	The publication bias related to data asymmetry was estimated by funnel plots and Egger's test. For further evaluation of heterogeneity in each model, we evaluated also radial plots, normal quantile-quantile (Q-Q) plots, and Baujat plots.	MM ("*Statistical Analysis*").
Additional analyses	16	Forest plots were created for each measured outcome to illustrate the effects of the different studies and the global estimation. In the random-effects models the selected studies and their outcomes are assumed to be a random selection from a larger population of studies. The random-effects models were evaluated for each effect size without and with moderator variables; in this last case, we obtained the corresponding mixed-effects models (one model for each moderator), where the coefficients from the fitted models estimate the relationship between the average true effect/outcome in the population of studies and the moderator variables included in the same models. The Knapp and Hartung method was used to adjust the standard errors of the estimated coefficients, which helps to account for the uncertainty in the estimate of residual heterogeneity. When moderators were included in the model, the Q_E-test was used to evaluate residual heterogeneity	MM ("*Statistical Analysis*").
**RESULTS**			
Study selection	17	An overview of our selection process for MR-related studies is presented in Figure 1A. An initial keyword search of the listed databases identified 11193 records. After duplicate removing and after excluded records in titles and abstracts, 43 full-text articles were assessed for eligibility (ESM - Table 4A). Of these, 9 were deemed eligible for inclusion in the meta-analysis. An overview of our selection process for US-related studies is presented in Figure 1B. An initial keyword search of the listed databases identified 17402 records. After duplicate removing and after excluded records in titles and abstracts, 17 full-text articles were assessed for eligibility (ESM - Table 4B). Of these, 12 were deemed eligible for inclusion in the meta-analysis.	Results ("*Literature search and study identification for MR*" and "*Literature search and study identification for intraoral US*"), Figure 1, ESM Table 4A-4B.
Study characteristics	18	Authors, Study type, Number of patiens, Tumor site, T, N, MR machine, US probe, Setting.	Results ("*Characteristics of included MR studies*", "*Characteristics of intraoral US included studies*"), Table 1, Table 2.
Risk of bias within studies	19	*Quality Assessment of Diagnostic Accuracy Studies* (QUADAS)-2 tool.	Results ("*Quality assessment and risk*

			of bias"), Table 3, Figure 3, Figure 5, ESM Figure 2, ESM Figure 4.
Results of individual studies	20	Correlation coefficient (*r*), Fisher's *r*-to-*z* transformation with 95% CI, diagnostic plots.	Results ("*Meta-analysis for studies related to MR*", "*Meta-analysis for studies related to intraoral US*"), Figure 2-5.
Synthesis of results	21	Concerning all the included MR studies the pooled *r* was 0.87 (95% CI from 0.82 to 0.92) whereas the pooled *r*-to-*z* transformed was 1.44 (95% CI from 1.02 and 1.85). For the MR studies (n = 8) that assumed TT as gold standard the pooled *r* was 0.87 (95% CI from 0.82 to 0.93) whereas the pooled *r*-to-*z* transformed was 1.46 (95% CI from 0.99 to 1.92).<br>Concerning all the included US studies the pooled *r* was 0.96 (95% CI from 0.94 to 0.97) whereas the pooled *r*-to-*z* transformed was 1.76 (95% CI from 1.39 and 2.13). For the US studies (n = 11) that assumed TT as gold standard the pooled r was 0.96 (95% CI from 0.95 to 0.98) whereas the pooled r-to-z transformed was 1.80 (95% CI from 1.40 to 2.20).	Results ("*Meta-analysis for studies related to MR*", "*Meta-analysis for studies related to intraoral US*").
Risk of bias across studies	22	*Grading of Recommendations, Assessment, Development and Evaluations* (GRADE) system.	Results ("*Quality assessment and risk of bias*"), ESM Table 5A-5B.
Additional analysis	23	In the random-effects model with only MR studies (n = 8) that assumed TT as gold standard (MR2 model), the I2 was 95.47% (95% CI from 86.74 to 98.57) and the H2 was 22.08 (95% CI from 7.54 to 70.09), with Q = 154.556 (P <0.001). The MR2 model resulted statistically significant (P <0.001). In the random-effects model with all intraoral US studies (n = 12) included (US1 model), the I2 was 93.56% (95% CI from 85.03 to 97.50) and the H2 was 15.53 (95% CI from 6.68 to 40.06), with Q = 170.884 (P <0.001). The US1 model resulted statistically significant (P <0.001). By entering the main variables (patient mean age and sex, tumor site, T, N, M, TT/DOI, probe) as moderators in the US1 model, patient sex (P = 0.006) and probes at 12 MHz (P = 0.005), 5-15 MHz (P = 0.002), 8-10 MHz (P = 0.003) and 5-10 MHz (P = 0.033) reached statistical significance.<br><br>In the random-effects model with only intraoral US studies (n = 11) that assumed TT as gold standard (US2 model), the I2 was 93.76% (95% CI from 84.69 to 97.67) and the H2 was 16.03 (95% CI from 6.53 to 42.95), with Q = 160.321 (P <0.001). The US2 model resulted statistically significant (P <0.001). By entering moderators in the US2 model as described above, patient sex (P = 0.005) and probes at 12 MHz (P = 0.005), 5-15 MHz (P = 0.002), 8-10 MHz (P = 0.003) and 5-10 MHz (P = 0.033) reached statistical significance.	Results ("*Meta-analysis for studies related to MR*", "*Meta-analysis for studies related to intraoral US*"), ESM Figure 1-4.
**DISCUSSION**			
Summary of evidence	24	Both MR and intraoral US were shown herein to have good correlation with histopathological findings. Nonetheless, the higher pooled r was seen in the intraoral US studies (0.96 vs. 0.87). Intraoral US was a better preoperative and intraoperative predictor of TT and DOI, particularly in early SCC located in the anterior part of the oral cavity.	Discussion.
Limitations	25	Potential weakness may have affected the review process (unintentional omission of papers and/or impossibility to retrieve some). Bias risks were also present in included studies, which influenced conclusion credibility, although they were globally considered using the	Discussion and "*Limitations of the*

		QUADAS-2 tool evaluation.<br><br>Execution modality and evaluation of the diagnostic exams were not always uniform among the considered papers: different equipment was used; the timing between imaging and histopathological examination, as well as between preoperative biopsy and imaging, were inconstant or unexplained. Furthermore, concerning intraoral US papers, only 2 of 11 considered DOI as a benchmark with histopathology and the remaining 9 used tumor thickness. Among the MR studies, 3 of 9 considered DOI and 6 tumor thickness.<br><br>Clinical heterogeneities influenced the outcomes. Although we conducted a meta-regression to detect whether clinical variables influenced the results, some variables could not be quantitatively detected. These included the diagnostic ability of radiologists and individual, unreported clinical variables. Inherent diversity among studies, small sample sizes, and other unpredictable biases are all possible limitations.	*meta-analysis*" subsection.
Conclusions	26	Higher pooled *r* was seen in the intraoral US studies (0.96 vs. 0.87), even though more research is needed to standardize the optimal PF. Considering the limitations of the studies included and the retrospective nature intrinsic to any meta-analysis, further prospective comparisons between intraoral US and MR in the same cohort of patients are strongly recommended. This will allow the identification of the best imaging technique to be specifically used in early and advanced oral SCC, possibly with a parallel reduction in terms of patient selection biases.	Discussion and "*Conclusions*" subsection.
**FUNDING**			
Funding	27	Not applicable	Not applicable (no grants for this study).

According to Moher D, Liberati A, Tetzlaff J, Altman DG, *The PRISMA Group (2009). Preferred Reporting Items for Systematic Reviews and Meta-Analyses: The PRISMA Statement*. PLoS Med 6(7): e1000097. doi:10.1371/journal.pmed1000097.

ESM Table 2. Full search strategy.

Database	Query	Time-point	Records
MEDLINE®/PubMed®	"(MR OR magnetic resonance) and (oral cavity OR head and neck) and (cancer OR tumor OR carcinoma) and (thickness OR depth of invasion OR depth of infiltration)"	01/01/1978 – 27/08/2019	11105
	"(US OR ultrasonography) and (oral cavity OR head and neck) and (cancer OR tumor OR carcinoma) and (thickness OR depth of invasion OR depth of infiltration)"	01/01/1978 – 27/08/2019	17322
Scopus®	"(MR OR magnetic resonance) and (oral cavity OR head and neck) and (cancer OR tumor OR carcinoma) and (thickness OR depth of invasion OR depth of infiltration)"	01/01/1978 – 27/08/2019	6
	"(US OR ultrasonography) and (oral cavity OR head and neck) and (cancer OR tumor OR carcinoma) and (thickness OR depth of invasion OR depth of infiltration)"	01/01/1978 – 27/08/2019	4
Science Citation Index® Expanded from Web of Science®	"(MR OR magnetic resonance) and (oral cavity OR head and neck) and (cancer OR tumor OR carcinoma) and (thickness OR depth of invasion OR depth of infiltration)"	01/01/1985* – 27/08/2019	78
	"(US OR ultrasonography) and (oral cavity OR head and neck) and (cancer OR tumor OR carcinoma) and (thickness OR depth of invasion OR depth of infiltration)"	01/01/1985* – 27/08/2019	74
Cochrane Library® via Wiley Online Library	"(MR OR magnetic resonance) and (oral cavity OR head and neck) and (cancer OR tumor OR carcinoma) and (thickness OR depth of invasion OR depth of infiltration)"	01/01/1978 – 27/08/2019	4
	"(US OR ultrasonography) and (oral cavity OR head and neck) and (cancer OR tumor OR carcinoma) and (thickness OR depth of invasion OR depth of infiltration)"	01/01/1978 – 27/08/2019	2

* The queries within the Science Citation Index® Expanded from Web of Science database are allowed from 01/01/1985.

A systematic literature search was performed in the following databases: MEDLINE/PubMed® (https://www.ncbi.nlm.nih.gov/pubmed), Scopus® (https://www.scopus.com/search/form.uri?display=basic), Science Citation Index® Expanded from Web of Science® (http://apps.webofknowledge.com/), ScienceDirect® (https://www.sciencedirect.com/), and Cochrane Library® (http://cochranelibrary-wiley.com/cochranelibrary/search). The queries were adapted to the conventions of each database, especially for the use of Boolean connectors in MEDLINE®/PubMed®, Scopus®,and Science Citation Index® Expanded from Web of Science®, as well as for medical subject headings (MeSH) in MEDLINE®/PubMed®. Reference lists of the pre-screened studies were manually checked, using an iterative approach.

ESM Table 3. Extracted data in each study assessed for eligibility.

**Extracted Data**	**Details**
Study Reference	Names and surnames of authors, year of publication.
Country	Country/countries in which the study was carried out.
Study design	Type of recruitment.
Gender	Percentage of male patients.
Age	Patient age reported in the study (as mean ± sd or median).
Patient number	Number of eligible patients, number of patients with oral cavity squamous cell carcinoma, number of excluded patients.
Pathology	Oral cavity site and subsites involved.
TNM	T stage and N stage of included patients.
Radiological instrumentation	MR machine (3T/1.5T); US probe frequencies.
Setting	Preoperative or intraoperative US measures.
Correlation coefficient (*r*)	Correlation coefficient between mean MR and US TT/DOI and hystopathological measures.

MR: magnetic resonance; US: ultrasonography; TT: tumor thickness; DOI: depth of invasion.

ESM Table 4A. Full text magnetic resonance-related articles excluded, not fitting eligibility criteria.

**Excluded studies**	**Main reason for exclusion**
Alsaffar et al. 2016	Pearson's *r* not evaluated
Angelelli et al. 2017	Pearson's *r* not evaluated, study design
Arakawa et al. 1996	Study design
Arya et al. 2014	Review article
Blatt et al. 2016	Review article
Boland et al. 2010	Tumor volume evaluated and not TT or DOI
Castelijns et al. 1991	Review article, no comparison with gold standard as reference.
Chew et al. 2007	Tumor volume evaluated and not TT or DOI
De Koning et al. 2019	Study design
Dhoot et al. 2015	Pearson's *r* not evaluated, no TT or DOI measured
Einspieler et al. 1991	Pearson's *r* not evaluated
Godeny et al. 2014	Review article
Hagiwara et al. 2012	Review article
Heissler et al. 1994	Tumor stage evaluated and not TT or DOI
Heppt et al. 1992	Pearson's *r* not evaluated, study design
Hoang et al. 1994	Review article
Holzapfel et al. 2008	Study design
Hu et al. 2015	Study design
Iwai et al. 2002	Study design
Kwon et al. 2015	Pearson's *r* not evaluated
Luccichenti et al. 2004	Review article
Lufkin et al. 1986	Study design
Palasz et al. 2017	Review article
Preda et al. 2006	Pearson's *r* not evaluated
Sarrion et al. 2015	Review article
Sigal et al. 1996	Review article
Singh et al. 2017	Study design
Takashima et al. 1993	Study design
Vidiri et al. 2007	Study design
Vidiri et al. 2019	Pearson's *r* not evaluated
Zhu et al. 2003	Study design
Zeng et al. 2003	Study design

ESM Table 4B. Full text intraoral ultrasonography-related articles excluded, not fitting eligibility criteria.

**Excluded studies**	**Main reason for exclusion**
De Koning et al. 2019	Study design
Helbig et al. 2005	Pearson's *r* not evaluated
Joshi et al. 2014	Study design and Pearson's *r* not evaluated
Nulent et al. 2017	Review article
Tarabichi et al. 2017	Pearson's *r* not evaluated

ESM Table 5A. Overall quality assessment for the magnetic resonance-related studies included in the meta-analysis, following the GRADE system.

Quality assessment							Summary of findings				
							No. of patients		Effect		Quality
**No. of studies**	**Design**	**Limitations**	**Indirectness of patients, intervention and comparator**	**Inconsistency**	**Imprecision**	**Other considerations**	**MRI**		**Relative (95% CI)**	**Absolute (95% CI)[2]**	
9 studies (487 adult patients)	5 prospective observational studies, 4 retrospective osservational studies	Some limitations exist[3]	NO	Serious[4]	NO	The QUADAS-2 suggested a high risk of bias for 3 studies[5]	487[1]	-	-	From 0.82 to 0.92	⊕⊕○○ Low

[1] Patients with OCSCC evaluated with preoperative MR. [2] 95% Confidence interval (CI) of thel pooled *r*. [3] Execution modality and evaluation of the diagnostic exams were not always uniform among the considered papers. [4] Wide variation in the *r* estimates, wide 95% CIs. [5] For more details, see Table 3.

Each domain was evaluated according to Ryan R, Hill S (2016) How to GRADE the quality of the evidence. Cochrane Consumers and Communication Group. Version 3.0 December 2016. Available on: http://cccrg.cochrane.org/author-resources (last access: June 15, 2018). The table structure and quality of evidence were showed according to Schünemann H, Brożek J, Guyatt G, Oxman A (2013) GRADE handbook for grading quality of evidence and strength of recommendations. The GRADE Working Group. Available on: https://gdt.gradepro.org/app/handbook/handbook.html (last access: May 19, 2018), and Guyatt GH, Oxman AD, Santesso N, Helfand M, Vist G, Kunz R, Brozek J, Norris S, Meerpohl J, Djulbegovic B, Alonso-Coello P, Post PN, Busse JW, Glasziou P, Christensen R, Schünemann HJ (2013) GRADE guidelines: 12. Preparing Summary of Findings tables - binary outcomes. J Clin Epidemiol 66:158-172 (doi: 10.1016/j.jclinepi.2012.01.012).

ESM Table 5B. Overall quality assessment for the intraoral ultrasonography-related studies included in the meta-analysis, following the GRADE system.

Quality assessment							Summary of findings				
							No. of patients		Effect		Quality
**No. of studies**	**Design**	**Limitations**	**Indirectness of patients, intervention and comparator**	**Inconsistency**	**Imprecision**	**Other considerations**	**US**		**Relative (95% CI)**	**Absolute (95% CI)[2]**	
12 studies (520 adult patients)	10 prospective observational studies, 2 retrospective observational studies	Some limitations exist[3]	NO	Serious[4]	NO	The QUADAS-2 outcome suggested a high risk of bias for 4 studies[5]	520[1]	-	-	From 0.94 to 0.97	⊕⊕○○ Low

[1] Patients with OCSCC evaluated with preoperative IOUS [2] 95% Confidence interval (CI) of the of thel pooled *r*. [3] Execution modality and evaluation of the diagnostic exams were not always uniform among the considered papers [4] Wide variation in the *r* estimates, wide 95% CIs. [5] For more details, see Table 3.

Each domain was evaluated according to Ryan R, Hill S (2016) How to GRADE the quality of the evidence. Cochrane Consumers and Communication Group. Version 3.0 December 2016. Available on: http://cccrg.cochrane.org/author-resources (last access: June 15, 2018). The table structure and quality of evidence were showed according to Schünemann H, Brożek J, Guyatt G, Oxman A (2013) GRADE handbook for grading quality of evidence and strength of recommendations. The GRADE Working Group. Available on: https://gdt.gradepro.org/app/handbook/handbook.html (last access: May 19, 2018), and Guyatt GH, Oxman AD, Santesso N, Helfand M, Vist G, Kunz R, Brozek J, Norris S, Meerpohl J, Djulbegovic B, Alonso-Coello P, Post PN, Busse JW, Glasziou P, Christensen R, Schünemann HJ (2013) GRADE guidelines: 12. Preparing Summary of Findings tables - binary outcomes. J Clin Epidemiol 66:158-172 (doi: 10.1016/j.jclinepi.2012.01.012).

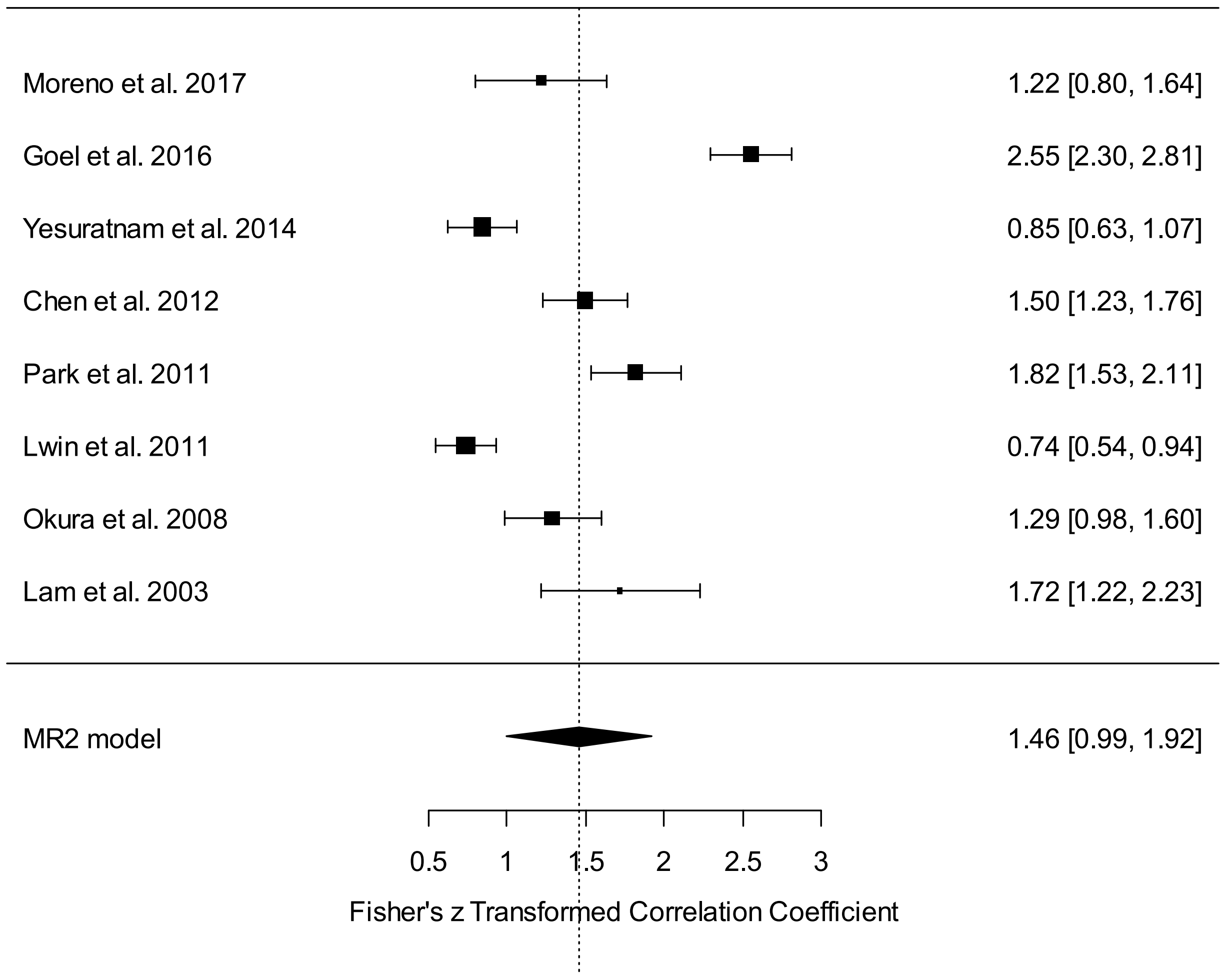

ESM Figure 1. Forest plot for *r*-to-*z* transformation in the random-effects model for included studies with tumor thickness evaluated by magnetic resonance (MR2 model).

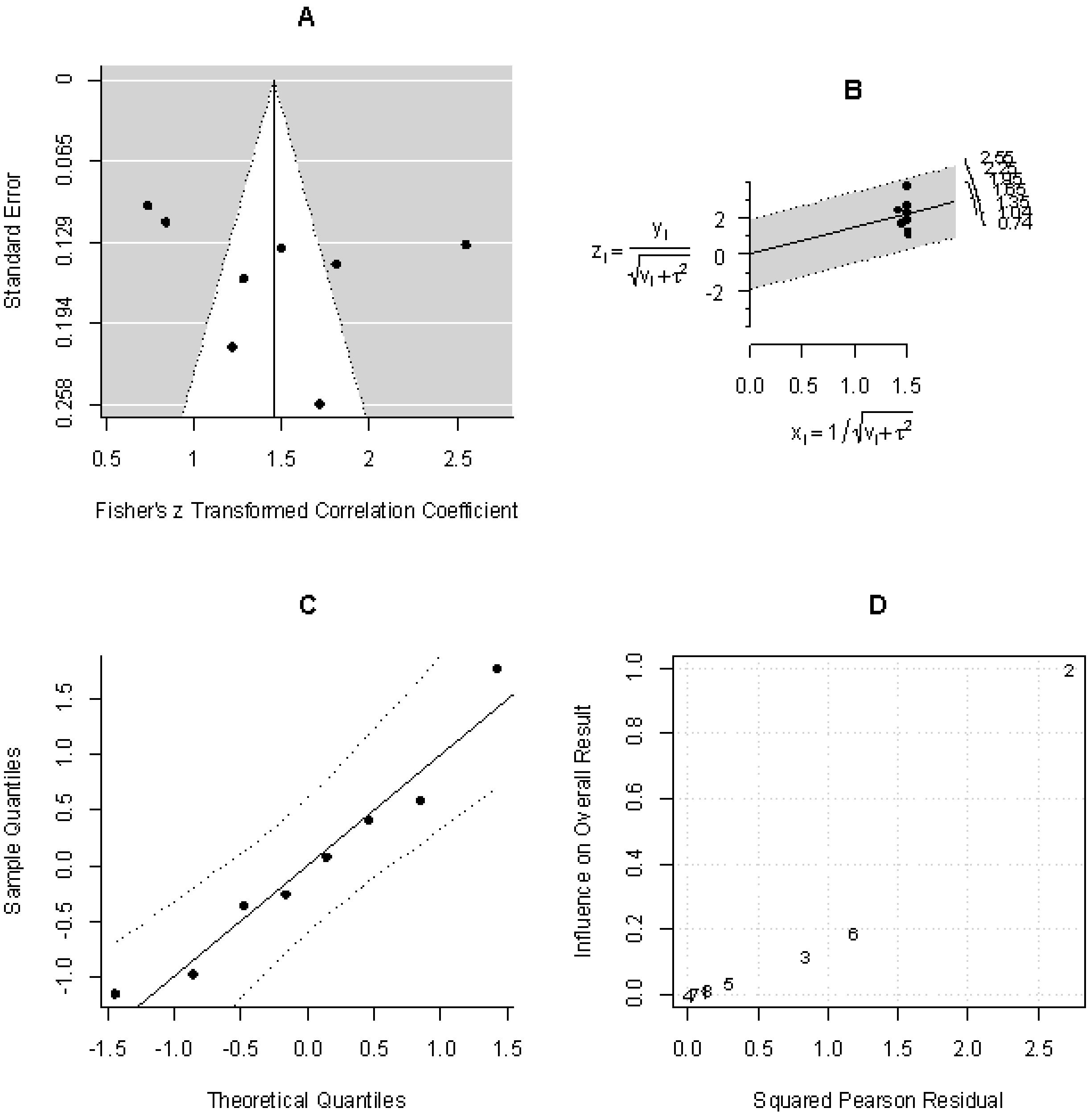

ESM Figure 2. Plots for evaluating publication bias and heterogeneity in the random-effects model for included studies with tumor thickness evaluated by magnetic resonance (MR2 model). A: funnel plot; B: radial plot; C: normal quantile-quantile plot; D: Baujat plot.

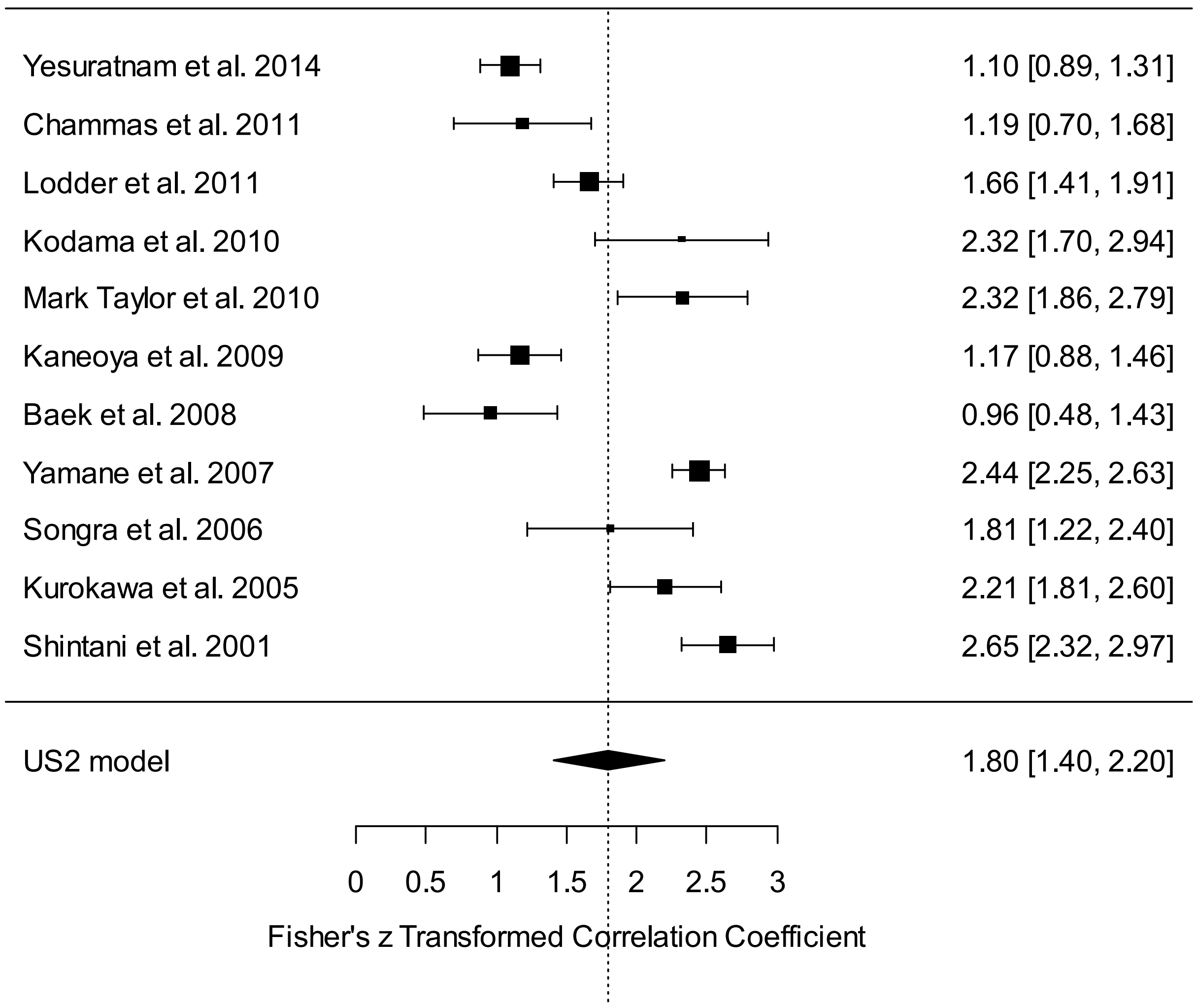

ESM Figure 3. Forest plot for *r*-to-*z* transformation in the random-effects model for included studies with tumor thickness evaluated by intraoral ultrasonography (US2 model).

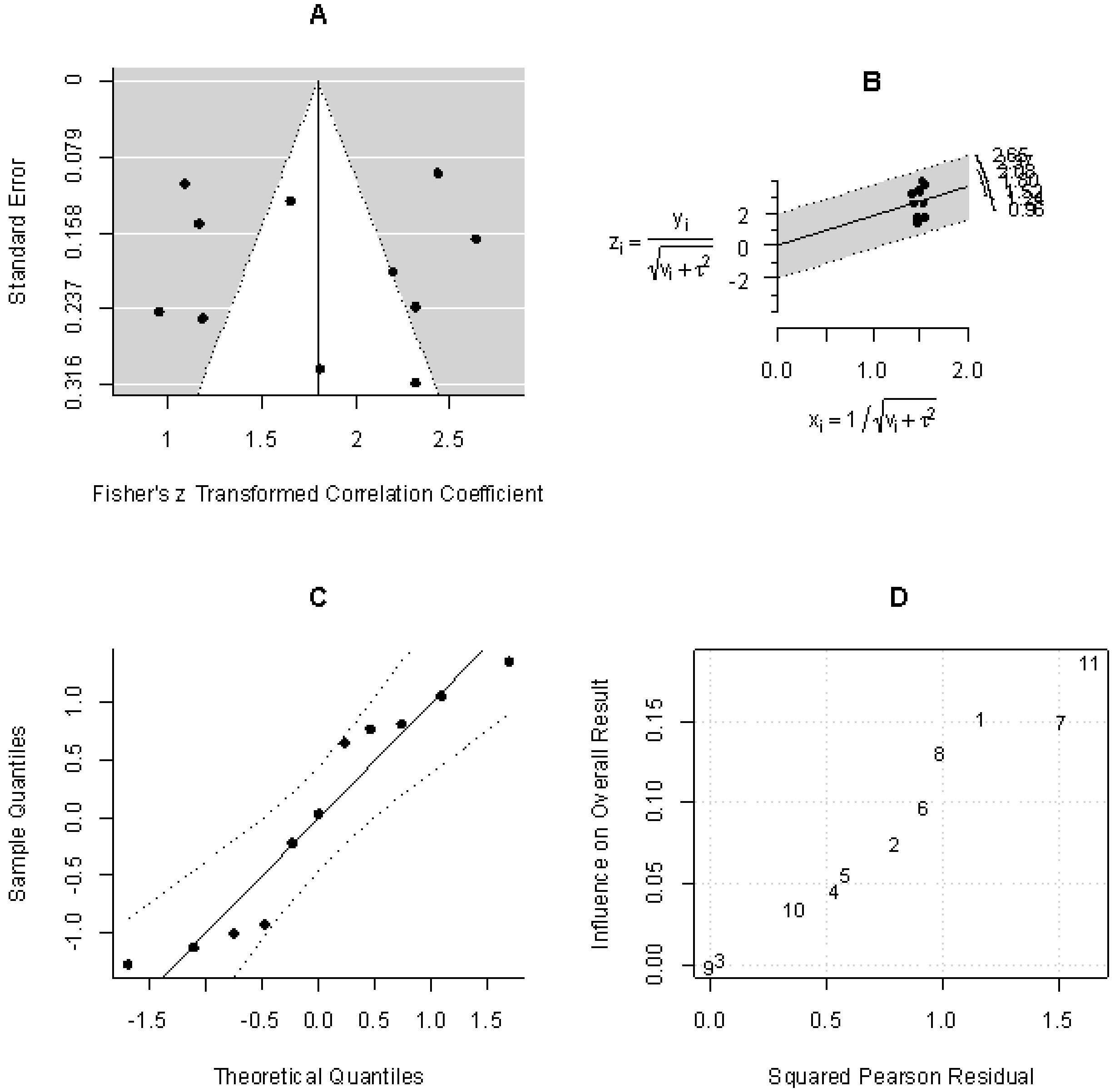

ESM Figure 4. Plots for evaluating publication bias and heterogeneity in the random-effects model for included studies with tumor thickness evaluated by intraoral ultrasonography (US2 model). A: funnel plot; B: radial plot; C: normal quantile-quantile plot; D: Baujat plot.
